# An evaluation of a canine dry wipe decontamination kit

**DOI:** 10.3389/fvets.2026.1770205

**Published:** 2026-02-12

**Authors:** Robert G. Buntz, Meghan K. Haselden, Kiran P. Badola, Julia L. Sharp, Kelly A. Mann, Brian France

**Affiliations:** 1 Department of Small Animal Primary Care, Midwestern University, Glendale, AZ, United States; 2 Mantel Technologies, Inc., Fort Collins, CO, United States; 3 Sharp Analytics LLC, Fort Collins, CO, United States; 4 Department of Clinical Sciences, Colorado State University, Fort Collins, CO, United States; 5 TDA Research, Inc., Golden, CO, United States

**Keywords:** canine, decontamination, dry decon, military working dog, multi-purpose canine

## Abstract

**Introduction:**

Decontamination of working dogs at the point of exposure is critical for military, law enforcement, and search and rescue operations in austere or hazardous environments where water may be scarce and conventional wet decontamination poses logistical and safety challenges. Prototype dry (waterless) wipe decontamination kits were evaluated for efficacy in removing a surrogate contaminant (GloGerm™) when used by canine handler teams performing total body field decontaminations. The prototype kit procedures employed a sequence of three microfiber towel types: (1) dry, to remove gross contaminants; (2) wet, saturated with a surfactant to clean away residual contaminants; and, (3) dry, to remove final contamination and residual surfactant. Several variables were investigated to refine the final design, test the effectiveness of decontamination at different anatomical locations on the canine, and evaluate the handlers’ success in removing the surrogate contaminant after reading the instructional pamphlet alone versus having the instructions and receiving in-person training on kit use.

**Methods:**

Three form factors (single towel, multi-towel small, and multi-towel large) and simulated contamination at three anatomical regions (back, inguinal, and forelimb) were tested by 64 canine handler teams randomized to “trained” (15-min verbal instruction plus pamphlet) or “untrained” (pamphlet only) groups.

**Results:**

Across all form factors and training groups, mean removal efficiencies were highest on the back (91.38% ± 0.18), followed by the forelimb (82.04% ± 0.26) and inguinal region (70.15% ± 0.31). No statistically significant differences were observed among the form factors or training variables. Average decontamination time was 9 min and 24 s (range = 3 min 4 s to 26 min 0 s).

**Discussion:**

Dry wipe decontamination can provide rapid and effective removal of contaminants without water, improving canine and handler safety and enabling early intervention at the point of exposure prior to additional wet decontamination or medical treatment when necessary. Personnel with minimal training can effectively conduct dry wipe decontamination.

## 1 Introduction

Emergency preparedness for the effective decontamination of a working dog (WD) at the point of exposure is a necessity for military, law enforcement, and search and rescue (SAR) canine teams. Their missions often occur in austere, limited resource environments, or in the immediate aftermath of man-made or natural disasters where hazardous materials exposures may be encountered unexpectedly (1). There is also the possibility of deliberate attacks using chemical, biological, or nuclear agents to disrupt protected human populations like the commuting public or military personnel. Limited protective equipment exists for WDs and available equipment may interfere with the dog’s ability to complete their job, increasing the risk of exposure (2).

Current canine decontamination procedures involve copious amounts of soap and water (i.e., “wet decontamination”) to remove contaminants (3–5). Access to safe, potable water is one of the most constrained resources in austere environments and creates a substantial logistical burden. Typically, the canine must be brought to a static wet decontamination lane location, delaying time from exposure to decontamination, allowing for transdermal absorption, and increasing human team members’ exposure risks via transference from the canine.

When a dog is exposed to hazardous or toxic agents, their fur or hair coat acts as the first line of defense. Materials and chemicals that contact the fur must penetrate through the coat before contacting and being absorbed through the underlying skin (6, 7). Use of high-pressure soap and water as the first step in decontamination can provide an avenue for fur penetration, transdermal absorption, and potential harm to a previously unaffected dog (8–10).

The use of soap and water has been shown to enhance the percutaneous absorption of lipophilic chemicals (6, 11) and repeated washing with soaps has been shown to damage the barrier function of the skin against external infectious or chemical agents (12). Additionally, repeated soap and water bathing may result in a pruritic inflammatory response, increasing the likelihood of licking and scratching behaviors and elevating the risk of absorption through skin penetration and oral contamination (13). SAR dogs will often work several weeks at the same disaster site, requiring repeated decontamination procedures which increases these risks during subsequent exposures (14).

Human procedures for entering a wet decontamination lane involve stripping off their outer layer of clothing, which removes up to 90% of the contaminant (15). As quadrupeds, canines may be exposed to significantly more contaminants than their human counterpart because they are at ground level and cannot remove their contaminated fur upon entering the decontamination lane (16). All of these factors, combined with normal dog behavior like tail wagging, shaking, and rolling on ground, cause significant cross contamination in the environment which increases risks to other animals and people in the area, and increases logistical management of contaminated water disposal. Furthermore, high pressure washing increases the risk of aerosolization of contaminants, which is exacerbated by the potential for a high contaminant load within the canine fur or hair coat (17, 18).

**Figure 1:**
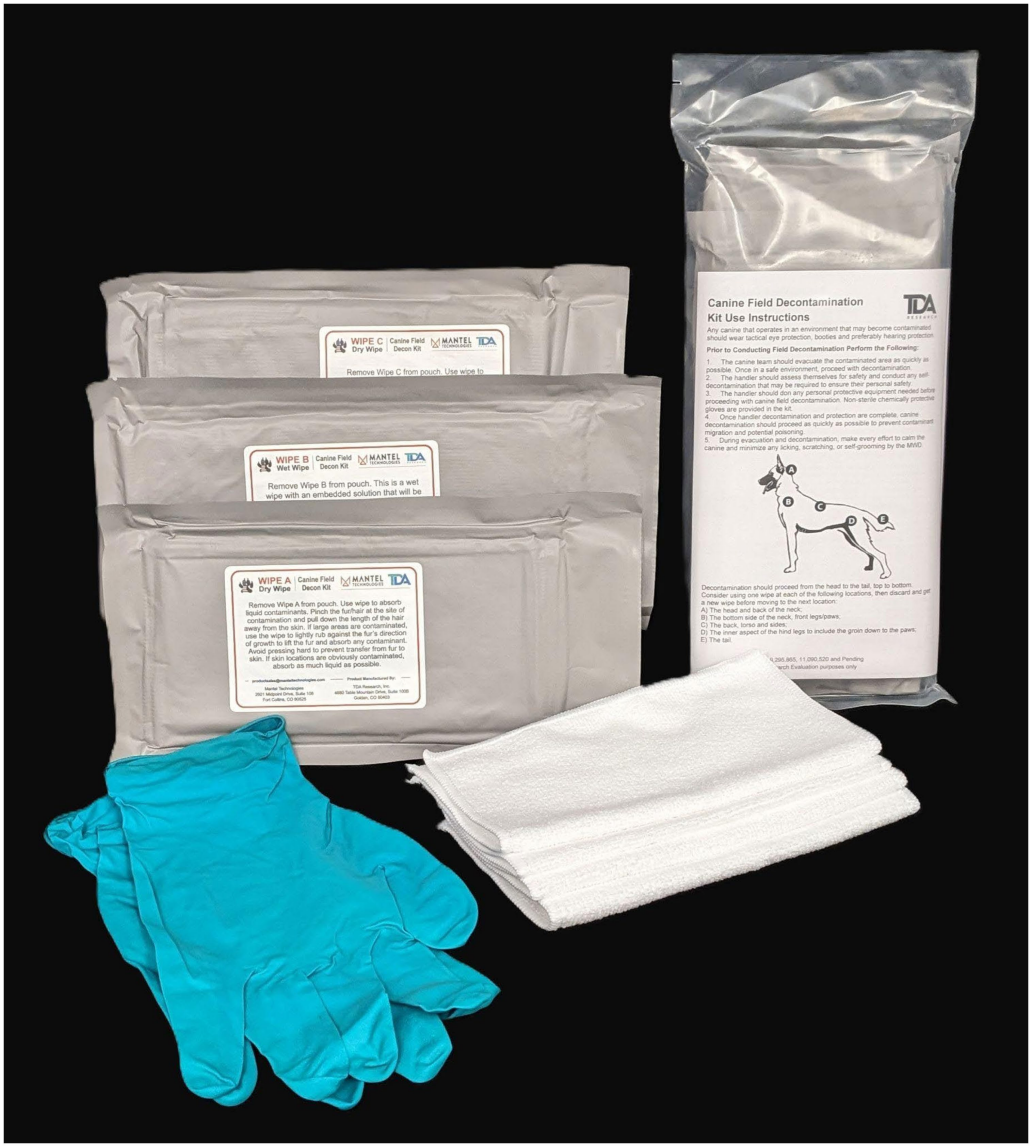
Prototype canine field decontamination kit. Three vacuum-sealed microfiber towel packages labeled Wipe A Dry Wipe, Wipe B Wet Wipe, and Wipe C Dry Wipe, protective non-sterile gloves, examples of the microfiber wipes, and the complete prototype canine field decontamination kit in overwrap packaging with the instruction sheet facing outwards. Not pictured: two slip leads, scissors, and M8 paper.

A dry (waterless) wipe decontamination kit and simple point of exposure procedures offer many benefits to current canine decontamination recommendations. Waterless decontamination with the prototype decontamination wipes used in this study and the “pinch and pull” technique was developed and studied under laboratory conditions for the removal of chemical warfare agents from canine cadaver skin/fur samples (7). The objective of this study was to evaluate the use and efficacy of prototype canine field decontamination kits (TDA Research, Inc., Golden, CO; Figure 1) in removing a safe, surrogate contaminant (GloGerm™ MIST Spray, Glo Germ Company, Moab, UT) from a working dog by their handler. This practical evaluation, in addition to live agent laboratory testing on canine fur samples (7), is important in assessing the kit form factors and effectiveness prior to potential adoption by military, federal and civilian working dog handlers, Chemical, Biological, Radiological, and Nuclear personnel, disaster response groups, first responders, and veterinary medical professionals.

**Figure 2:**
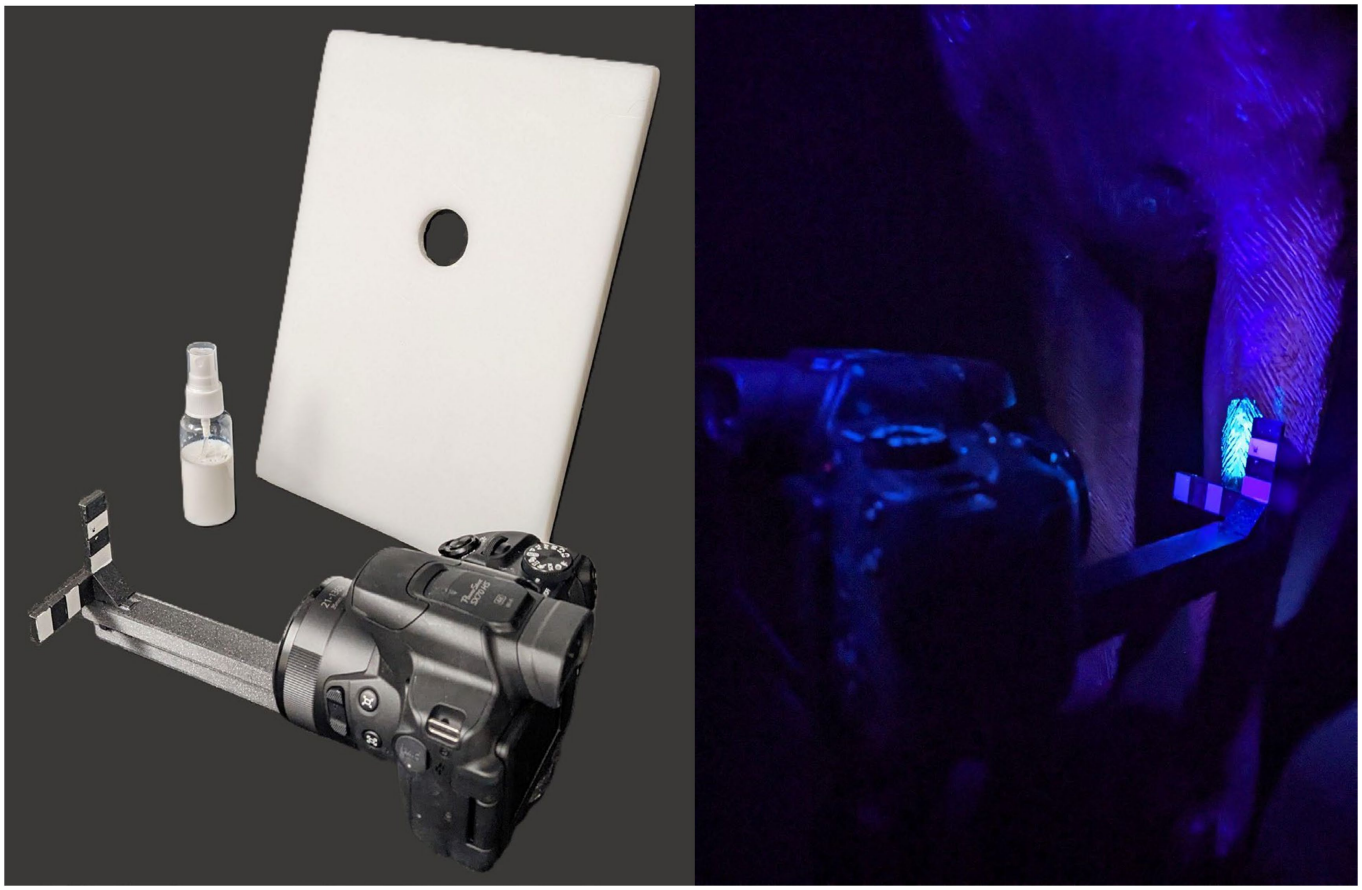
Application of contaminant. GloGerm™ MIST Spray, plastic stencil for uniform distribution of simulant, and camera with standoff for uniform image acquisition (left). Photo acquisition after “contamination” with GloGerm™ on the forelimb (right).

Live agent studies using chemical warfare and other life-threatening contaminants are important in assessing the effectiveness of decontamination equipment and procedures; however, they have several limitations. As these contaminants cause severe life-threatening effects, they cannot safely be used on active WDs by their handlers. Live agent testing is limited to controlled environments where biosafety personnel can safely conduct tests on cadaver fur and tissue samples. Combining the results from laboratory live agent studies and on-dog simulant contaminant removal studies allows for a more complete assessment of the effectiveness and limitations of canine decontamination procedures (19). Practical evaluation with canine handler teams encompasses important aspects such as canine restraint and decontamination on a non-static target, canine behaviors toward a novel stimulus of this decontamination type, difference in fur length and composition across individual dogs, non-ideal angle and positioning for the handler to reach differing anatomic locations during decontamination, and feasibility of decontaminating the entirety of a dog (as opposed to smaller laboratory fur samples). The outcome of interest in this study was the evaluation of laboratory-developed decontamination techniques in real world environments, the percentage of simulant removed helps us understand the challenges and variables faced during decontamination events. The following variables were assessed in this study:

*Anatomical Location/Hair Type Effectiveness*: Three anatomical locations were chosen based on their differences in hair types and in handler access during decontamination: (1) the back (dorsum) with its longer primary or guard hairs and ease of handler access, (2) the inguinal (inner thigh) region with its shorter, thinner secondary hairs and more difficult to reach location, and (3) the forelimb below the elbow with its shorter guard hairs.
*Form Factor Effectiveness*: Three form factors were evaluated in this study, looking primarily at the wipe size and number of wipes needed to decontaminate a canine. The form factors tested included: (1) “single towel,” a single ‘large’ microfiber towel (40 × 40-cm) stored in vacuum-sealed packages were used for each step (e.g., Wipe A Dry Wipe, Wipe B Wet Wipe, and Wipe C Dry Wipe; Figure 1), (2) “multi-towel small,” multiple (5 each) small towels (20 × 20-cm) in each vacuum-sealed package, and (3) “multi-towel large,” multiple (3 each) large towels (40 × 40-cm) in each vacuum-sealed package. This study compared the decontamination effectiveness among these form factors, allowing for evidence-based selection of the form factor for optimization of the final kit through downselection.
*Trained Versus Untrained Effectiveness*: A one-page instructional pamphlet was included in each prototype canine field decontamination kit. In order to assess the decontamination effectiveness of the instructions, a trained group (provided with both the instructional pamphlet and verbal instruction by the research team) was compared to an untrained group (provided an instruction pamphlet only).

Handlers included in the study encompassed a wide variety of working dog groups including military, special operations, law enforcement, and civilian SAR groups, with the purpose of capturing how persons of different backgrounds and experience levels would be able to effectively employ the prototype canine field decontamination kit. Additionally, handler feedback was collected and used to assess the kit’s function in proposed real-world scenarios and to optimize future kit design.

## 2 Materials and methods

### 2.1 Participants

Institutional Animal Care and Use Committee (IACUC) and Institutional Review Board (IRB) approval was obtained from Colorado State University prior to the initiation of this study. Participants were WDs and their handlers from military, law enforcement, or SAR backgrounds. Permissible breeds were German, Belgian (e.g., Groenendael, Tervuren, Malinois, Laekenois), and Dutch Shepherds, or mixes of the aforementioned breeds. The canines were 1–10 years of age, either male or female, neutered or intact. The canines could be excluded from the IACUC protocol based on aggressive temperament, active dermatologic conditions, or subjective exclusion by their attending veterinarian, unit commander, or supervisor for any reason without explanation or fear of retribution.

### 2.2 Application of contaminant

The simulant contaminant used was a commercial product, GloGerm™. GloGerm™ is available in powder, spray and gel formulations which are well known in the safety training community and have been employed by other working dog research teams (3, 14, 16, 19, 20). In this study, colorless, GloGerm™ MIST Spray droplets were used to simulate a potentially lethal toxin exposure in a safe non-threatening environment for the canine handler team. The simulant was applied in the presence of the handler, however the GloGerm™ was not visible to the unaided eye once applied, making the exact location of the contamination less obvious. GloGerm™ MIST Spray (Figure 2) was applied topically to each canine at three anatomical locations: dorsal midline behind the shoulder blades (“back”); the inner aspect of either hindlimb above the knee where the abdomen meets the inner thigh (“inguinal”); and the cranial aspect of either forelimb between the carpus and elbow (“forelimb”). A plastic barrier with a cutout (circle, 3.2-cm diameter) for the application of the GloGerm™ (Figure 2) was utilized to prevent overspray and standardize the area of GloGerm™ application. The plastic template was held against the dog and GloGerm™ was sprayed once over the cutout to produce consistent application size at all three locations on the individual dog as well as for each canine participant in the study. Before the application of GloGerm™, a pre-contamination photo was obtained under ultraviolet (UV) illumination in a darkened room. After the application of GloGerm™, photographic images using UV illumination were obtained in a darkened room before and after the handler performed the decontamination procedure using the provided prototype kit. A camera standoff (Figure 2) provided for a consistent 13-cm distance between the camera lens and the dog when each photograph was taken as well as a consistent 1-cm reference measurement within each photograph in order to quantify the area of simulant fluorescence during analysis. After initial photography and simulant application, the handler was instructed to conduct a full canine decontamination while being observed and timed by a research team member. After the handler completed the decontamination procedure, the locations where simulant had been applied were re-photographed and analyzed to determine effectiveness of the kit materials and the procedure to remove the simulant.

### 2.3 Prototype canine field decontamination kit and wipe procedures

The waterless, non-reactive decontamination procedure recommended in the prototype canine field decontamination kits uses a sequence of three microfiber towel types: (1) dry to initially remove gross contaminants from the fur (e.g., package labeled Wipe A Dry Wipe); (2) wet which is saturated with a surfactant to clean away residual contaminants on the fur (e.g., package labeled Wipe B Wet Wipe); and, (3) dry to remove final contamination and residual surfactant (e.g., package labeled Wipe C Dry Wipe; Figure 1). The procedures recommended in the kit instructions were designed to transfer contaminants from the canine’s fur to the microfiber towels while keeping contaminants off and away from the canine’s skin.

**Figure 3:**
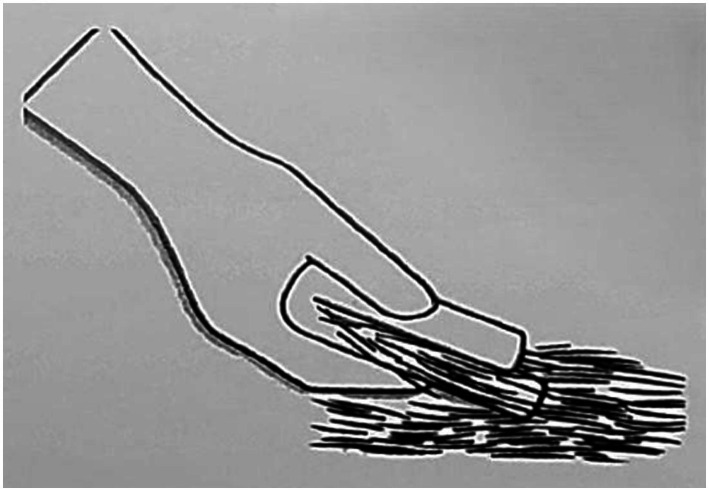
Pinch and pull technique used with all wipes to remove contaminant without pushing it deeper to contact the skin.

This study was conducted in small groups at individual canine handler team locations based on their schedule and availability. Each participating canine handler team was randomly assigned to one of three possible form factors: single towel, multi-towel small, and multi-towel large. Each handler was randomized into a trained or untrained category and one of the three possible form factor subcategories. The trained group participants received approximately 15 min of interactive instruction from a research team member in addition to the kit use instruction sheet and specific instruction on how to implement the “pinch and pull” technique (Figure 3). The untrained group participants received just the kit use instruction sheet to read at their own pace with no additional interactive training from the research team. All participants received identical instruction sheets.

### 2.4 Image analysis

Analysis of photos was performed with FIJI image software (based on ImageJ, originally developed by the National Institutes of Health). The “particle count” tool was used with a mask subtraction technique to objectively quantify the quantity of fluorescent particles removed by the handlers using the prototype canine field decontamination kits. Three photos from the same dog and same anatomical location (one pre-contamination photo, one post-contamination photo, and one post-decontamination photo) were captured and uploaded to FIJI. In order to measure particle count, these three images were cropped to include only the simulant contaminated region of interest (e.g., excluded the 1-cm fiducial for photographic scaling). These images were then compiled into a ‘stack’ on FIJI and converted from color to 8-bit black and white images. From there, the threshold of all images was adjusted at once. The ‘pre’ image was selected to view as the threshold is adjusted, as there should be no contamination visible on the ‘pre-contamination’ image. The threshold was adjusted so that there were no visible particles (e.g., dust and dander can fluoresce under UV light) in the ‘pre-contamination’ images, and that same threshold was applied to all the images in the stack. This allowed the images to be analyzed by comparing the quantified particle count for the ‘post-contamination’ and ‘post-decontamination’ images. The fluorescent particle counts were calculated by selecting ‘analyze particles’ on FIJI. This computes a fluorescence particle count for each image, and these values were recorded for further analysis to calculate the percent particle count decontamination. Similarly, the FIJI software “size” tool was used with a mask subtraction technique to objectively measure the area (cm^2^) of fluorescent simulant reduction in the same images. The results from the area reduction method did not differ substantially from the particle count method and were excluded from this report in support of continued use of the quantitative particle count method in this and future studies.

### 2.5 Mitt form factor

Early in this investigation, a mitt form factor was evaluated. Despite positive feedback from end users, the mitt form factor was found to be underperforming compared to the other form factors (single towel, multi-towel small, multi-towel large) and presented logistical burdens in both manufacturing and utilization. Due to the static position on the hand, the surface area in direct contact with the dog’s coat was limited. Only the area between the fingertips and the palm on one side of the mitt made direct contact with the fur during the “pinch and pull” method and the back of the mitt never contacted the fur. The additional thickness of the mitt increased the size and weight of the kit which was an important consideration raised in feedback from all end users. Due to poor early performance, difficulties in manufacturing, and limited adaptability for use with different personal protective equipment (PPE) and individual hand sizes, along with the increase in kit size/weight, the mitt form factor was excluded from further canine handler team evaluation and from data analysis.

### 2.6 Statistical analysis

Descriptive statistics were computed for the percentage of decontamination for both “size” (area; cm^2^) calculated with the FIJI software area tool and “particle count” (FIJI software image subtraction tool) reduction considered across all variables (trained versus untrained, towel form factors, anatomical location). Histograms and dot plots were constructed to visualize the distribution of percentage decontamination “particle count” and “size.” For each anatomical location and form factor combination, the distribution of the number of dogs in the quartile categories was compared between the trained and untrained groups using Fisher’s Exact Test. Similarly, for each location and training group, the distribution of the number of dogs in the quartile categories was compared among the kit combinations using Fisher’s Exact Test. When differences in either percentage “particle count” or “size” decontamination between training groups were not found, training groups were combined to compare the kit combinations at each location using Fisher’s Exact Test. A significance level of 0.05 was used for all tests of significance. Data was also evaluated using the above methods as “pass” versus “fail,” with <75% of contaminant removed denoting a failure, in order to consider areas that were overlooked by the handler or inadequately decontaminated by the wipe procedures and not substantially contacted with the decontamination wipes.

## 3 Results

A total of 64 canine handler teams evaluated the prototype canine field decontamination kits. Ten participants each in the untrained single towel and untrained multi-towel large categories and eleven participants each in the trained single towel, untrained and trained multi-towel small, and trained multi-towel large categories. The means and standard deviations of percentage of decontamination based on particle count for all kit combinations and both training groups are in Table 1. For all form factor combinations and trained and untrained groups, the mean percentage decontamination particle count for the back was higher than the forelimb and the inguinal locations.

**Table 1:** The percentage of decontamination based on particle count for all kit combinations and both training groups.

Percentage of decontamination based on particle count			
Category	Back	Forelimb	Inguinal
Single towel trained	98.61% (±2.81)	72.10% (±39.4)	81.54% (±23.64)
Single towel untrained	87.28% (±23.09)	85.21% (±24.58)	61.83% (±36.98)
Multi-towel small trained	99.69% (±0.75)	84.87% (±19.19)	62.90% (±35.17)
Multi-towel small untrained	88.51% (±22.67)	72.36% (±31.06)	82.38% (±30.60)
Multi-towel large trained	78.52% (±32.67)	70.09% (±34.24)	74.32% (±32.02)
Multi-towel large untrained	95.48% (±13.55)	83.81% (±19.80)	64.88% (±35.32)

**Figure 4:**
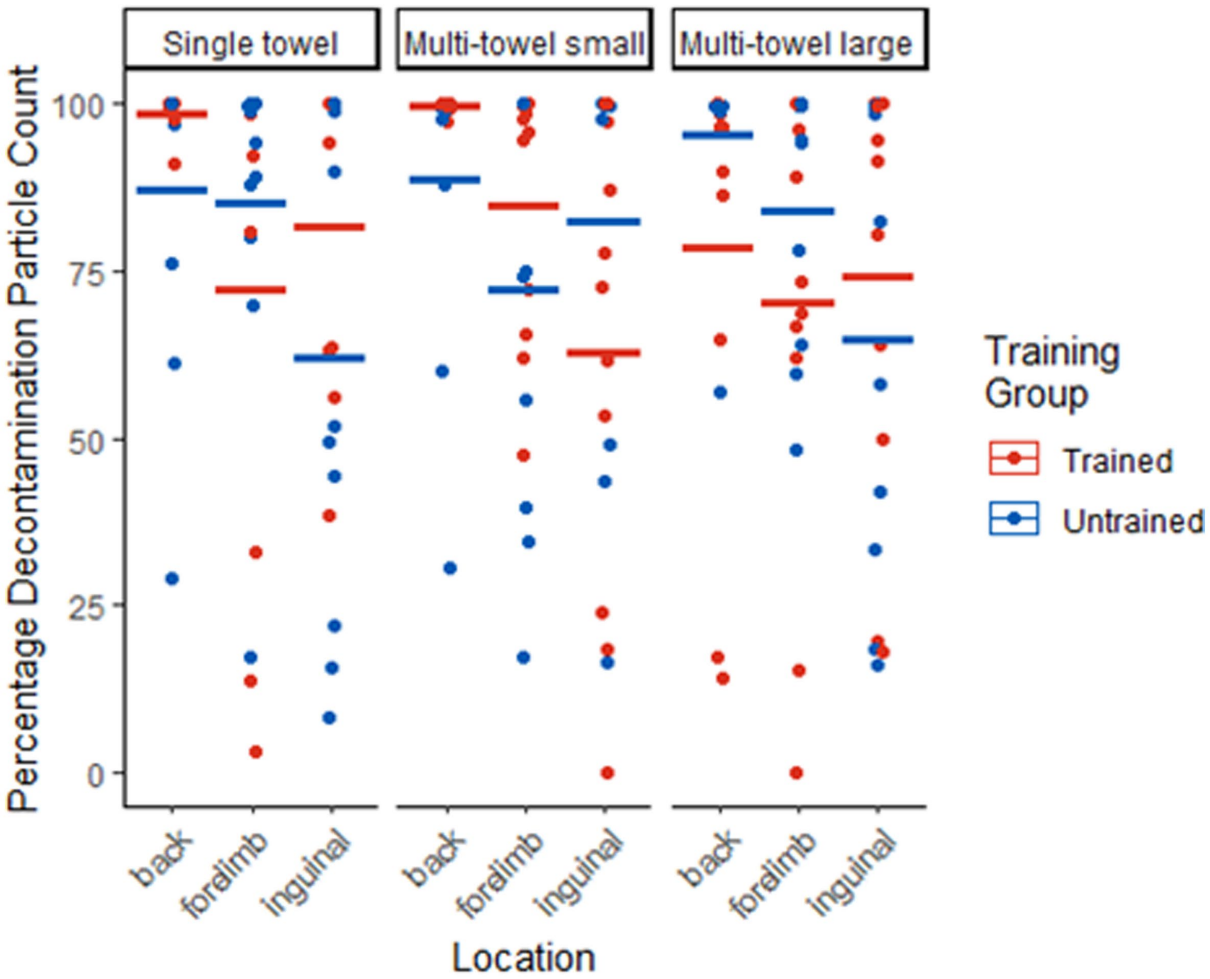
Dot plot of individual decontamination results (percent reduction in particle count) across anatomical locations (back, forelimb, inguinal) and form factors (single towel, multi-towel small, multi-towel large). Means represented by horizontal lines.

**Figure 5:**
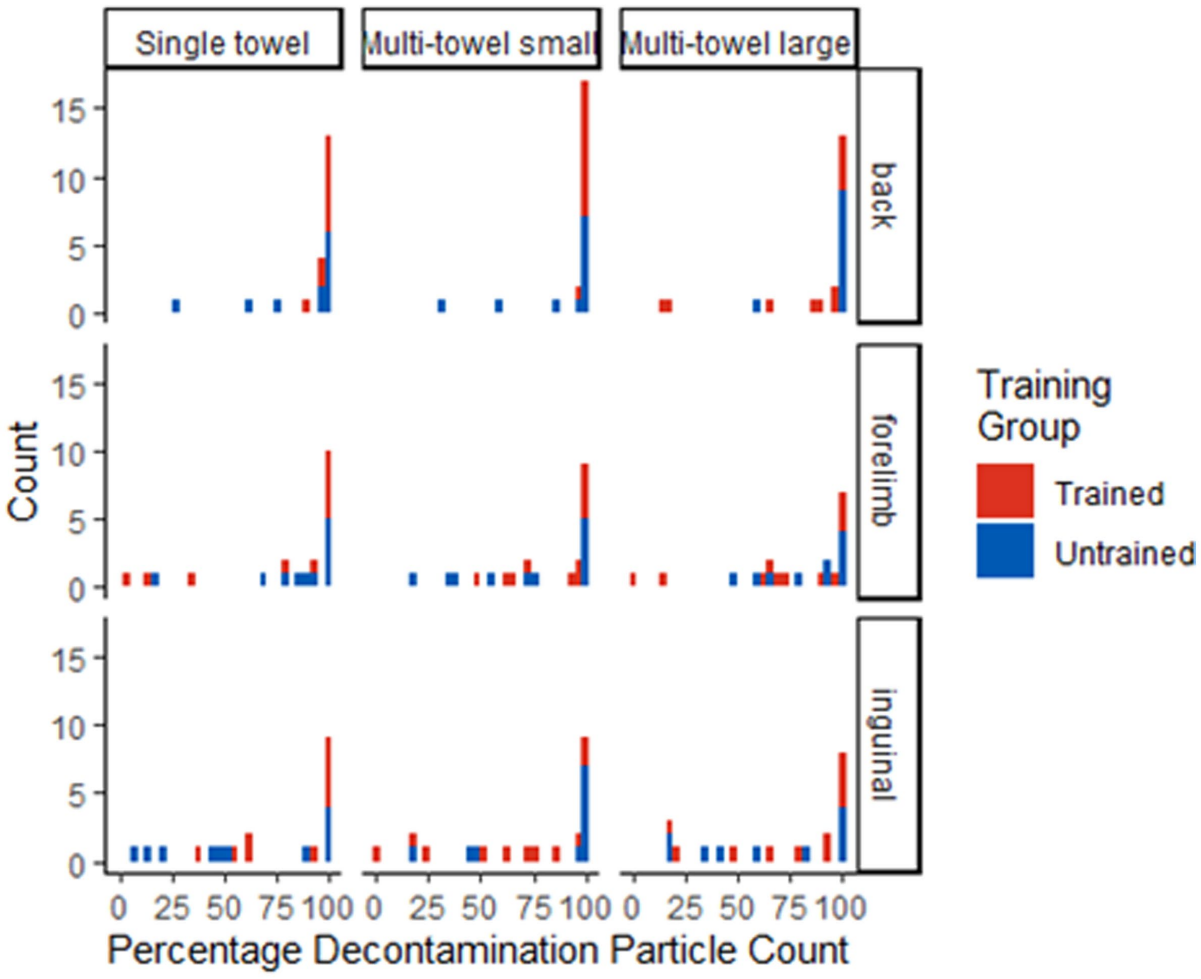
Histogram depicting the decontamination results (percent reduction in particle count) across anatomical locations (back, forelimb, inguinal) and form factors (single towel, multi-towel small, multi-towel large) separated into quartiles.

The number of observations that had 75–100% particle count reduction was largest for all kit form factors, training categories, and all anatomical locations (Table 2; Figures 4, 5). For all locations and both training groups, there was not sufficient evidence that the distribution of dogs across the percentage particle count decontaminated quartiles differed among the three kit combinations (*p*-value range 0.157 and 1.000; Table 3). There was not sufficient evidence that the distribution of dogs exceeding the 75% pass criteria differed among the three kit combinations (*p*-value range 0.387 to 1.000; Table 3). Also, there was not sufficient evidence that the distribution of dogs across the percentage particle count decontaminated quartiles differed between the trained and untrained groups (p-value range 0.113 to 1.000; Table 4) or that the distribution of dogs exceeding the 75% pass criteria differed between the trained and untrained groups (p-value range 0.387 to 1.000; Table 4).

**Table 2:** The number of observations of percentage of particle count reduction for all kit form factors, training categories, and all anatomical locations.

Form factor	Training category	Location	Percentage particle count reduction			
			0–25%	25–50%	50–75%	75–100%
Form factor A	Trained (*N* = 10)	Back				10
		Forelimb	2	1		7
		Inguinal		1	3	6
	Untrained (*N* = 11)	Back		1	1	9
		Forelimb	1		1	9
		Inguinal	3	2	1	5
Form factor C	Trained (*N* = 11)	Back				11
		Forelimb		1	3	7
		Inguinal	3		3	5
	Untrained (*N* = 11)	Back		1	1	9
		Forelimb	1	2	2	6
		Inguinal	1	2		8
Form factor D	Trained (*N* = 11)	Back	2		1	8
		Forelimb	2		4	5
		Inguinal	2	1	1	7
	Untrained (*N* = 10)	Back			1	9
		Forelimb		1	2	7
		Inguinal	2	2	1	5

**Table 3:** Fisher’s exact test *p*-values comparing the distribution of percentage particle count quartiles among the form factors for each location and training group found no significant differences.

Comparison among form factor particle count			
Location	Training group	*p*-value	Above 75% decontamination threshold *p*-value
Back	Trained	0.157	0.091
	Untrained	1	1
Forelimb	Trained	0.193	0.552
	Untrained	0.757	0.408
Inguinal	Trained	0.489	0.742
	Untrained	0.87	0.449

**Table 4:** Fisher’s exact test *p*-values comparing the distribution of percentage particle count quartiles between trained and untrained groups for all locations and each form factor found no significant differences.

Comparison between trained and untrained groups particle count			
Location	Form factor	*p*-value	Above 75% decontamination threshold *p*-value
Back	Single towel	1	0.476
	Multi-towel small	0.476	0.476
	Multi-towel large	0.724	0.587
Forelimb	Single towel	0.672	0.635
	Multi-towel small	1	1
	Multi-towel large	0.329	0.387
Inguinal	Single towel	0.316	0.67
	Multi-towel small	0.113	0.387
	Multi-towel large	0.906	0.67

## 4 Discussion

### 4.1 Anatomical location effectiveness

The highest decontamination efficacy based on mean particle count reduction was noted on the back (91.38% ± 0.18). The guard hairs at this anatomical location are longer, which allows for more efficient performance of the recommended “pinch and pull” decontamination technique, are the most easily accessible for the handler, and have more intrinsic protective qualities when compared to other hair types. The forelimb and inguinal areas had less contaminant removed across the form factors and both training groups. The forelimb had a mean fluorescent particle count reduction of 82.04% ± 0.26. Due to the short guard hairs at this anatomical location, decontamination using the “pinch and pull” method is difficult and less practical in a moving canine. Some handlers disregarded the recommended “pinch and pull” technique in the instructions and utilized a “circumferential rub” technique. Observationally, this may have improved their efficiency of GloGerm™ removal and time spent decontaminating the limbs. This technique may be added to future kit instructions and dry wipe decontamination procedures. When spraying GloGerm™ on the shorter or thinner haired areas on the limbs of some dogs, the GloGerm™ droplets penetrated the fur and directly contacted the skin. This is noted because the goal is to avoid skin contact with the contaminant since this is the point at which the canine could become poisoned. Also, contaminated skin is anticipated to be the most challenging location to decontaminate because the overlying hair may block direct physical contact with the wipe.

The inguinal area had a mean reduction of 70.15% ± 0.31 for fluorescent particle count across all form factors and training groups. This represents failed decontamination of this anatomical region based on the 75% cutoff used in the study. The hairs in this anatomical location are considered undercoat, lacking the protective quality of guard hairs present in other areas. The inguinal area is also the most difficult to reach and most easily overlooked by the handler. Additionally, the hairs vary in length and density and in some individual dogs there was GloGerm™ penetration to the skin. Some members of the canine community hypothesize the inguinal area is less likely to be contaminated due to its anatomically protected location. However, discussion with subject matter experts (SME) during military decontamination training events provided observational evidence to the contrary. For example, a dog walking in tall grass or lying down on a surface will easily have droplets transfer from the contaminated vegetation or surface to their ventrum. Peer-reviewed literature has also shown the ventral surfaces of the dog are more likely to be contaminated and more likely to be missed during wet decontamination (16). While the Perry et al. (16) study focuses on environmental contamination from standing liquid hazards which may differ from the dispersion of a chemical warfare agent, the findings reinforce the need for proper decontamination of ventral anatomical areas. In other decontamination training events, lifting of the canine onto a table or tailgate improved the handler’s ability to access and decontaminate the ventrum and inguinal areas of the canine. For these reasons, kit use instructions and training recommendations must highlight frequently encountered human errors in order to optimize decontamination efficacy. Proper restraint techniques to ensure the physical safety of the handler and other personnel assisting with canine decontamination as well as proper wear of PPE must be emphasized to avoid contaminant transference to additional personnel and equipment. Finally, sedation may be required in cases of medically symptomatic or overtly aggressive canines to allow a handler to implement the “pinch and pull” and rub or wipe techniques in difficult to reach and often overlooked anatomical areas.

### 4.2 Form factor effectiveness

There was no statistically significant difference between the single towel, multi-towel small, and multi-towel large form factors in percentage of fluorescent particle reduction, percentage of area reduction, or quartile reduction categories (e.g., 0–25, 26–50, 51–75, 76–100%). Combining objective data collection with handler and SME feedback informed several kit improvement recommendations. For example, the multi-towel large form factor was much larger in size and weight, and there was not a significant improvement in handler decontamination efficacy, therefore this kit form factor was excluded from production consideration. The larger towels were often noted to be unwieldy as handlers tried to use the clean portions of the towel without cross contamination of the dog or themselves from unclean portions of the towel. Handlers preferred to use and discard multiple small towels. The single towel and multi-towel small kits both had advantages: the single towel was the most compact and lightweight of all the evaluated form factors, giving it a significant advantage in handler feedback; however, one towel was not adequate for whole body decontamination (e.g., quickly covered with dirt, loose fur) and shared similar cross contamination issues described for large towels. Mass animal decontamination in disaster response preparedness is a growing area of concern. The single large towel form factor may be the most beneficial in mass casualty scenarios where kits could be provided to pet owners prior to going through wet decontamination. This would allow for earlier contaminant removal, decreased wicking of contaminants to the dog’s skin, and decreased cross contamination. For individual canine handler use at the point of exposure, the multi-towel small form factor has the advantage of using a separate towel for specific regions of the body to avoid tracking contamination to clean and sensitive areas. The multi-towel small form factor was more favorably viewed by medical personnel for this reason. Training handlers in a decontamination pattern based on anatomical region may improve the percentage removal of contaminants. For example, use the first towel on the head and neck region and discard the towel, use the second towel on the back and discard, etc.

### 4.3 Trained versus untrained effectiveness

There was no statistically significant difference found between the “trained” and “untrained” variables. The lack of significant difference between these two groups provides evidence that the dry wipe decontamination procedures are intuitive and that the written instructions were clear, easy to understand, and easy to implement by working dog handlers.

### 4.4 Average time for kit use

The average time for decontamination using the prototype canine field decontamination kit was 9 min 24 s (range = 3 min 4 s to 26 min 0 s) across all handler backgrounds, trained and untrained groups, and kit form factors. However, handlers were not wearing full Mission Oriented Protective Posture (MOPP) gear or PPE. This average time can be used as a baseline for planning purposes in a hasty decontamination or limited exposure scenario. A significant time difference is expected for decontamination in lethal environments with personnel operating in Joint Service Lightweight Integrated Suit Technology overgarments or Advanced Chemical Protective Garments.

### 4.5 Inter-study comparison

Waterless decontamination with the prototype canine field decontamination kits used in this study and the “pinch and pull” technique was developed and studied under laboratory conditions for the removal of sulfur mustard (HD; blister agent) and venomous agent X (VX; nerve agent) from canine cadaver skin/fur samples (7). The results of handler GloGerm™ decontamination efficacy on the canine back were similar to the live agent laboratory decontamination results. The lower mean particle count reduction on the back by the handler (91.38%) versus mean reduction of VX (97.9%) and HD (99.6%) from canine cadaver fur samples may be attributed to the research team’s visual observation of 11% of handlers omitting an area when decontaminating the entire dog versus conditions in a controlled laboratory setting where the location of contaminant was known. This inter-study comparison lends strength to the necessity and ability of a canine handler to initiate decontamination at the point of exposure. Additionally, the levels of effective decontamination demonstrated by the handlers in this study provides evidence that untrained individuals can effectively utilize the dry wipe decontamination method in a realistic scenario.

### 4.6 Limitations

The training guidelines were created early in the study design based on published canine decontamination literature and laboratory studies. As the study progressed, discussions with professional working dog personnel and observations of handler kit use and effectiveness led to productive insights. This information was not implemented during the study to avoid affecting data collection. However, new recommendations were discussed with handlers and SMEs after data collection at each testing site with favorable feedback. Additionally, handlers were not required to wear full MOPP gear or PPE during decontamination procedures. In real-world scenarios when wearing this gear, it is expected that performing decontamination procedures and canine handling will increase in difficulty, increasing the time required to fully decontaminate a WD. Individual handler experiences with other decontamination methods was unknown in this study. Each handler received instructions as described but then were allowed to conduct the decontamination procedure to their satisfaction without further guidance from the research team. Future experimental designs could evaluate handler-related variables like prior training and deployment experience as well as the conditions necessary to improve efficiency and efficacy in trained decontamination procedures. This study included only German, Belgian, and Dutch Shepherd breeds. Future research should include breeds with a wider variety of fur textures and thicknesses.

### 4.7 Advantages of field decontamination

Using the canine field decontamination kit allows for debulking prior to and in conjunction with soap and water decontamination protocols. Dry wipe decontamination leverages the intrinsic protective mechanisms of the canine’s fur coat, and prevents initial wetting of the coat which has been shown to cause transport of the contaminant to the skin and transdermal absorption. Field decontamination compliments current water-based decontamination protocols by reducing aerosolization risks, reducing excessive contaminated water, and reducing environmental and cross contamination through its more controlled collection of contaminants within the microfiber towels. The contaminated wipes can then be disposed of as a solid waste whereas soap and water wash decontamination methods create a significant logistical challenge to dispose of up to 40 L of contaminated water per canine (8). The dry wipe decontamination technique limits contact with the skin and the prototype kit contains a proprietary surfactant (not a soap) proven to be non-irritating for both canine and human skin. These factors limit the impact of decontamination on the natural skin barrier, making dry wipe decontamination more suitable when repeated decontamination procedures are necessary (e.g., daily SAR work in a contaminated environment) (13, 21).

The prototype canine field decontamination kit has been designed and tested for single handler use, reducing stress on the canine and preventing additional personnel exposures. It allows for canine decontamination to be performed at the point of contamination and, when possible, away from large crowds and loud equipment. By reducing stress of the canine, a more thorough decontamination can be performed. This reduces the human health hazards associated with an incomplete decontamination of the canine, re-exposure of personnel to contaminants, PPE suit tear risks, and/or physical injury to the canine or personnel.

In a mass contamination scenario, using the prototype canine field decontamination kit, handlers can simultaneously decontaminate their canines as opposed to a one at a time system of a wet soap and water lane. This allows early decontamination, early medical intervention, decreased cross contamination, and overall improvement in canine and human outcomes and survivability. In civilian canine contamination scenarios, field decontamination can be performed by untrained personnel like pet owners or disaster relief volunteers.

### 4.8 Future research and recommendations

The canine field decontamination kit is non-reactive, surfactant-based, and may be effective for a variety of chemical, biological, and nuclear contaminants, but because of their inherent dangers, individual contaminant types must be specifically tested in controlled laboratory studies. Individual training at the unit-level as well as complex multiagency events should include hands-on decontamination scenarios using GloGerm™ or other traceable simulants to allow handlers to practice a variety of decontamination techniques while they experience and discover their own lessons learned (e.g., circumferential rub technique for the forelimb, elevation of the canine to reach the ventrum, a step-wise approach to the multiwipe kits, commonly missed anatomical areas) while improving their confidence, efficiency and efficacy.

## 5 Conclusion

The use of a canine field decontamination kit can provide rapid and effective removal of contaminants without water and fills a needs gap in current canine decontamination protocols. Personnel with minimal training can effectively conduct dry wipe decontamination. A compact, lightweight, waterless kit allows the canine handler to initiate decontamination at the point of exposure, decreasing canine absorption and transference risks, and allowing for decontamination prior to transportation to a wet decontamination site or medical care. Implementation of field decontamination procedures can significantly improve the prognosis for contaminated canine survival and lessen the risk of dangerous toxin transference to their human teammates.

## Data Availability

The raw data supporting the conclusions of this article will be made available by the authors, without undue reservation.

## Author’s note

This manuscript uses “military working dog” as the encompassing term for MWDs, Multi-Purpose Canines, and other categories of working dogs employed by DOD components and interagency partners.
